# Expanding the etiologic spectrum of spastic ataxia syndrome: chronic infection with human T lymphotropic virus type 1

**DOI:** 10.1007/s13365-020-00932-2

**Published:** 2021-03-22

**Authors:** Karolina af Edhom, Christer Lidman, Tobias Granberg, Graham P. Taylor, Martin Paucar

**Affiliations:** 1 Department of Neurology, Danderyd’s Hospital, Stockholm, Sweden; 2 Department of Infectious Diseases, Karolinska University Hospital, Stockholm, Sweden; 3 Department of Neuroradiology, Karolinska University Hospital, Stockholm, Sweden; 4 Department of Clinical Neuroscience, Karolinska Institutet, Stockholm, Sweden; 5 Section of Virology, Department of Infectious Disease Imperial College London, London, UK; 6 Department of Neurology, Karolinska University Hospital, Stockholm, Sweden

**Keywords:** Ataxia, HTLV-1, Proviral load

## Abstract

Human T-lymphotropic virus type-1 (HTLV-1) is a neglected infection most often associated with an indolent process. However, a subset of HTLV-1 seropositive patients face the risk to develop life-threatening T-cell lymphoma/leukemia, or the highly disabling and incurable HTLV1-associated myelopathy/tropical spastic paraparesis (HAM/TSP). Over the years, other complications to HTLV-1 have been proposed and debated intensely. One of these, although rare, associations include cerebellar ataxia occurring most often in Japanese patients with manifest HAM/TSP. Here we present a HTLV-1 seropositive patient from the Middle East featuring a slowly progressive cerebellar syndrome with cerebellar atrophy but not evidence of spastic paraparesis. In addition, this patient suffered from autoimmune conditions such as Sjögren’s syndrome and vitiligo which are putatively associated with HTLV-1.

Infection with human T cell lymphotropic virus type 1 (HTLV-1) is in most cases indolent; however, some patients develop adult T cell leukemia, associated with poor prognosis, or the highly disabling and incurable HTLV-1-associated myelopathy/tropical spastic paraparesis (HAM/TSP) (Verdonck et al. 2007; Cooper et al. 2009). HTLV-1 is an endemic infection in Southern Japan, Iran, South America, the Caribbean basin, West Africa, and among aborigines in Australia (Verdonck et al. 2007). There are no established biomarkers to predict complications in HTLV-1; however, the percentage of peripheral blood mononuclear cells (PBMCs) harboring the provirus, called proviral load (PVL), and beta-2 microglobulin (β2M) in serum are surrogate biomarkers. Associations with neurological syndromes other than HAM/TSP have been claimed, including neuropathy, motor neuron disease (Araujo et al. 2019), as well as cerebellar ataxia (Iwasaki 1990; Kira et al. 1993; Gracia et al. 1995; e-1 to e-6). In the majority of reported cases, ataxia occurred in Japanese patients with HAM/TSP (Iwasaki 1990; Iwanaga 1993; Kira et al. 1993; e1, e-2, e-4, e-6). Here, we present an Iranian HTLV-1 positive patient with a cerebellar syndrome, elevated β2M in serum, and elevated neopterin and CXCL10 in cerebrospinal fluid (CSF).

## Case presentation

Written consent was obtained for this case report, approved by the Ethics Committee in Stockholm. A 68-year-old woman from Mashhad, Iran, was referred for a progressive movement disorder. Onset of neurological symptoms was at age 59, 6 years before HTLV-1 infection was diagnosed as a result of contact tracing. The patient reported insidious onset of gait difficulties, obstipation, and urinary urgency. During follow-up, falls started to occur motivating the use of a walker. Her comorbidities consisted of hypertension, fibromyalgia, right shoulder impingement, asthma, Sjögren’s syndrome, vitiligo, tremor, and a history of surgery for ileus. Examination at age 62 revealed postural and action hand tremor, dysmetria, axial difficulties, and inability to perform tandem gait. The patient had brisk patellar reflexes (3+), normal Achilles reflexes, and absence of Babinski’s sign. In addition, her muscle tone, strength, and sensation to touch and pin prick were normal. Vibration sense in her malleoli and proprioception were impaired with subsequent abnormal Romberg’s test; the retropulsion test was normal. Her altering hand movements were irregular; bradykinesia was absent. The patient also displayed hypermetric saccades. Her Scale for the Assessment and Rating of Ataxia score rose from 7.5 to 9.5 and Instituto de Pesquisa Clinica Evandro Chagas HAM disability scale, from 8 to 10, over 5 years.

**Fig. 1:**
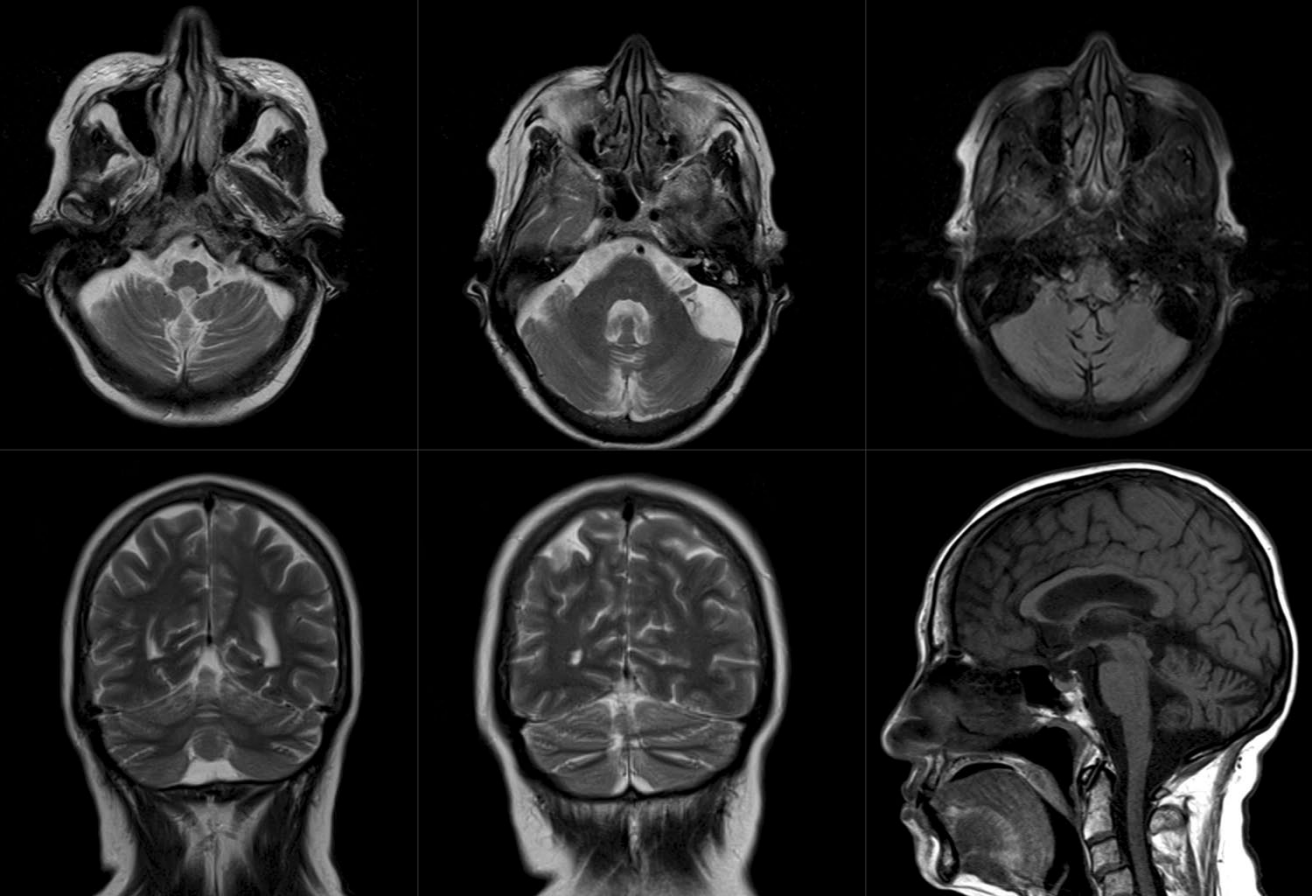
Neuroimaging in a HTLV-1-positive patient with ataxia. Brain magnetic resonance imaging of the reported patient at age 62 years of age showing diffuse subtle atrophy of the cerebellar hemispheres and vermis. Top row: axial T2-weighted (left and middle) and T2-weighted fluid-attenuated inversion recovery (right). Bottom row: coronal T2-weighted (left and middle) and sagittal T1-weighted (right) images

Neuroimaging demonstrated cerebellar atrophy (Fig. 1) but not evidence of widespread white matter abnormalities (WMA); lumbar spondylosis was evident, but there were no abnormalities in the spinal cord. Electromyography, electroneurography, sensory (SEP), and motor-evoked potentials (MEP) were normal except for incidental carpal tunnel syndrome.

The most common genetic ataxias, syphilis, Lyme’s and Whipple’s disease, and autoimmune ataxias were ruled out (Supplementary document). Thus, onconeuronal antibodies, antibodies for celiac disease, and against GAD were absent. PVL in blood was initially 0.54% and 1.4% when repeated whereas serum β2M increased from 2.5 to 4.4 mg/L (ref < 2.0) over 4 years. In the CSF, white cell count, albumin concentration, and neurofilament light protein (NfL) concentration were normal, with no malignant cells, protein 14-3-3 or oligoclonal bands. However, CSF PVL (4 HTLV DNA copies/15 cells), neopterin 3.7 pg/mL (reference 2–3 pg/mL), and CXCL10 249.1 pg/mL (reference 115–160.8 pg/mL) were high.

Computed tomography of her chest and abdomen showed an enlarged thyroid gland but the patient was euthyroid; a biopsy aspirate demonstrated colloid cells only. Mammography was initially normal but at age 68, she was diagnosed with a breast tumor treated with surgery and chemotherapy. Oral steroids were given with chemotherapy, but this did not alleviate the patient’s motor symptoms.

## Discussion

So far, 20 HTLV-1 seropositive patients displaying ataxia have been reported, mostly in Japan. All but two had manifest HAM/TSP, which suggests that pyramidal signs may overshadow subtle cerebellar signs and nystagmus (Kira et al. 1993). Only in three cases, cerebellar signs preceded pyramidal signs (e3, e4, e7). In our case, urgency, obstipation, hyperreflexia, and impaired vibration suggest an incipient myelopathy even though she does not have spastic paraparesis. Ataxia among HTLV-1-positive patients is associated with variable cerebellar atrophy (40% of cases) and rarely with WMA, pontocerebellar, or spinal cord atrophy (Kira et al. 1993 e-2–e-6). Insidious onset, slow course, and long latency between ataxia onset and diagnosis of breast cancer (9 years) argue strongly against paraneoplastic cerebellar degeneration (PCD). Furthermore, absence of onconeuronal antibodies and the pattern of CSF alterations add support to the exclusion of PCD.

β2M is elevated in some hematological malignancies and renal impairment but is also used as a surrogate marker for HAM/TSP (e-7). In this case, β2M and PVL increased during follow-up and PVL in the CSF was high. Of note, determining PVL in the CSF is challenging due to the low number of leukocytes in CSF.

Importantly, neurological complications are associated with high PVL (> 1 HTLV DNA copy per 100 PBMC, > 1%). β2M is a component of the major histocompatibility complex class 1 and a key element in immediate immune response. As a consequence of the inflammatory reaction, chronic inflammation of the nervous system may occur in predisposed individuals, leading to permanent neurological dysfunction. The pattern of interleukin elevation in the CSF has been reported in HAM/TSP patients with very slow progression (Sato et al. 2018). Clinical stratification with patterns of neopterin and CXCL10 predict response to treatment with steroids (Sato et al. 2018). Thus, very slow progression and mild elevations of neopterin and CXCL10 may explain the lack of benefit for treatment with oral steroid, albeit at a lower dosage than used in HAM/TSP, in our case. Raised serum β2M and HTLV-1 PVL > 1% in blood and higher in CSF, as well as elevated concentrations of neopterin and CXCL10 in CSF support the notion of a putative association between cerebellar ataxia and HTLV-1. Our findings have to be replicated in other ataxia and spastic ataxia cases associated with HTLV-1; in addition, more neuropathological studies are warranted in order to characterize the neuroanatomic correlations to motor dysfunction.

### Electronic supplementary material

Below is the link to the electronic supplementary material.

Supplementary file1 (ODT 6 KB)

Supplementary file2 (DOC 59 KB)

## Appendix: Authors

**Table:**

Name	Location	Role	Contribution
Karolina af Edholm, MD	Danderyd’s Hospital, Stockholm	Author	Analysis and interpretation of data, drafting and revising the manuscript
Christer Lidman, MD, PhD	Karolinska University Hospital and Karolinska Institutet, Stockholm	Author	Patient care, interpretation of clinical data; revising the manuscript
Tobias Granberg, MD, PhD	Karolinska University Hospital and Karolinska Institutet, Stockholm	Author	Interpretation of neuroimaging data; revising the manuscript
Graham Taylor, MD, DSc	Section of Virology, Department of Infectious Disease, Imperial College London, United Kingdom	Author	Interpretation of clinical data; selected CSF analyses, drafting and revising the manuscript
Martin Paucar, MD, PhD	Karolinska University Hospital and Karolinska Institutet, Stockholm	Author	Study concept and design; patient care, interpretation of clinical data; drafting and revising the manuscript
