# Supplementary material for: Expanding the etiologic spectrum of spastic ataxia syndrome: chronic infection with human T lymphotropic virus type 1
Source: J Neurovirol. 2021 Mar 22;27(2):345–7. doi: 10.1007/s13365-020-00932-2 (PMC8192349; doi:10.1007/s13365-020-00932-2)
Supplement: Supplementary file 2 — Supplementary file2 (DOC 59 KB) [file 13365_2020_932_MOESM2_ESM.doc]

**Case report**

**Expanding the etiologic spectrum of spastic ataxia syndrome: chronic infection with human T lymphotropic virus type 1**

Karolina af Edhom^1^, MD; Christer Lidman^2^, MD, PhD; Tobias Granberg^3,4^, MD, PhD; Graham P. Taylor^5^, MD, DSc; and Martin Paucar^4,6^, MD, PhD

^1^Department of Neurology, Danderyd’s Hospital, Stockholm, Sweden

^2^Department of Infectious Diseases, Karolinska University Hospital, Stockholm, Sweden

^3^Department of Neuroradiology, Karolinska University Hospital, Stockholm, Sweden

^4^Department of Clinical Neuroscience, Karolinska Institutet, Stockholm, Sweden

^5^Section of Virology, Department of Infectious Disease Imperial College London, United Kingdom

^6^Department of Neurology, Karolinska University Hospital

**Key words:** Ataxia, HTLV-1, proviral load

Word count for the paper: 965; word count for title: 17

Figures: 1

References: 8

Supplementary document: 1

**Corresponding Author:**
Martin Paucar, Department of Neurology, Karolinska University Hospital Huddinge, Sweden.
Phone +46 858580000

Carolina af Edholm karolina.afedholm-arvidsson@sll.se

Christer Lidman christer.lidman@ki.se

Tobias Granberg [tobias.granberg@ki.se](mailto:tobias.granberg@ki.se)

Graham P. Taylor g.p.taylor@imperial.ac.uk

Martin Paucar [martin.paucar-arce@sll.se](mailto:martin.paucar-arce@sll.se)

**Financial Disclosures:**

Carolina af Edholm- Reports no disclosures

Christer Lidman- Reports no disclosures

Tobias Granberg- Reports no disclosures

Graham P. Taylor- Reports no disclosures

Martin Paucar- Reports no disclosures

**Funding information**

MP’s and TG’s research is supported by Region Stockholm.

Infection with human T-cell lymphotropic virus type 1 (HTLV-1) is in most cases indolent, however, some patients develop adult T-cell leukemia, associated with poor prognosis, or the highly disabling and incurable HTLV-1-associated myelopathy/tropical spastic paraparesis (HAM/TSP) (1, 2). HTLV-1 is an endemic infection in Southern Japan, Iran, South America, the Caribbean basin, West Africa, and among aborigines in Australia (1). There are no established biomarkers to predict complications in HTLV-1, however, the percentage of peripheral blood mononuclear cells (PBMCs) harboring the provirus, called proviral load (PVL), and beta-2 microglobulin (β2M) in serum are surrogate biomarkers. Associations with neurological syndromes other than HAM/TSP have been claimed, including neuropathy, motor neuron disease (3), as well as cerebellar ataxia (4-7, e1-e6). In the majority of reported cases, ataxia occurred in Japanese patients with HAM/TSP (4, 6, 7, e-1, e-2, e-4, e-6). Here we present an Iranian HTLV-1 positive patient with a cerebellar syndrome, elevated β2M in serum and elevated neopterin and CXCL10 in cerebrospinal fluid (CSF).

**Case presentation**

Written consent was obtained for this case report, approved by the Ethics Committee in Stockholm. A 68-year-old woman from Mashhad, Iran, was referred for a progressive movement disorder. Onset of neurological symptoms was at age 59, six years before HTLV-1 infection was diagnosed as a result of contact tracing. The patient reported insidious onset of gait difficulties, obstipation, and urinary urgency. During follow-up, falls started to occur motivating the use of a walker. Her comorbidities consisted of hypertension, fibromyalgia, right shoulder impingement, asthma, Sjögren’s syndrome, vitiligo, tremor and a history of surgery for ileus. Examination at age 62 revealed postural and action hand tremor, dysmetria, axial difficulties and inability to perform tandem gait. The patient had brisk patellar reflexes (3+), normal Achilles reflexes and absence of Babinski’s sign. In addition, her muscle tone, strength and sensation to touch and pin prick were normal. Vibration sense in her malleoli and proprioception were impaired with subsequent abnormal Romberg’s test, the retropulsion test was normal. Her altering hand movements were irregular, bradykinesia was absent. The patient also displayed hypermetric saccades. Her Scale for the Assessment and Rating of Ataxia score rose from 7.5 to 9.5 and Instituto de Pesquisa Clinica Evandro Chagas HAM disability scale, from 8 to 10, over 5 years.

Neuroimaging demonstrated cerebellar atrophy (Figure 1) but not evidence of widespread white matter abnormalities (WMA); lumbar spondylosis was evident but there were no abnormalities in the spinal cord. Electromyography, electroneurography, sensory (SEP) and motor evoked potentials (MEP) were normal except for incidental carpal tunnel syndrome.

The most common genetic ataxias, syphilis, Lyme’s and Whipple’s disease and autoimmune ataxias were ruled out (Supplementary document). Thus, onconeuronal antibodies, antibodies for celiac disease and against GAD were absent. PVL in blood was initially 0.54 % and 1.4% when repeated whereas serum β2M increased from 2.5 to 4.4 mg/L (ref <2.0) over four years. In the CSF, white cell count, albumin concentration and neurofilament light protein (NfL) concentration were normal, with no malignant cells, protein 14-3-3 or oligoclonal bands. However, CSF PVL (4 HTLV DNA copies/15 cells), neopterin 3.7 pg/mL (reference 2-3 pg/mL) and CXCL10 249.1 pg/mL (reference 115-160.8 pg/mL) were high.

Computed tomography of her chest and abdomen showed an enlarged thyroid gland but the patient was euthyroid, a biopsy aspirate demonstrated colloid cells only. Mammography was initially normal but at age 68, she was diagnosed with a breast tumor treated with surgery and chemotherapy. Oral steroids were given with chemotherapy but this did not alleviate the patient’s motor symptoms.

**Discussion**

So far, 20 HTLV-1 seropositive patients displaying ataxia have been reported, mostly in Japan. All but two had manifest HAM/TSP, which suggests that pyramidal signs may overshadow subtle cerebellar signs and nystagmus (7). Only in three cases, cerebellar signs preceded pyramidal signs (e3, e4, e7). In our case, urgency, obstipation, hyperreflexia and impaired vibration suggest an incipient myelopathy even though she does not have spastic paraparesis. Ataxia among HTLV-1 positive patients is associated with variable cerebellar atrophy (40% of cases) and rarely with WMA, pontocerebellar or spinal cord atrophy (7, e2-e6). Insidious onset, slow course and long latency between ataxia onset and diagnosis of breast cancer (9 years) argue strongly against paraneoplastic cerebellar degeneration (PCD). Furthermore, absence of onconeuronal antibodies and the pattern of CSF alterations add support to the exclusion of PCD.

β2M is elevated in some hematological malignancies and renal impairment but is also used as a surrogate marker for HAM/TSP (e-7). In this case, β2M and PVL increased during follow-up and PVL in the CSF was high. Of note, determining PVL in the CSF is challenging due to the low number of leukocytes in CSF.

Importantly, neurological complications are associated with high PVL (>1 HTLV DNA copy per 100 PBMC, >1%). β2M is a component of the major histocompatibility complex class 1 and a key element in immediate immune response. As a consequence of the inflammatory reaction, chronic inflammation of the nervous system may occur in predisposed individuals, leading to permanent neurological dysfunction. The pattern of interleukin elevation in the CSF has been reported in HAM/TSP patients with very slow progression (8). Clinical stratification with patterns of neopterin, and CXCL10 predict response to treatment with steroids (8). Thus, very slow progression and mild elevations of neopterin and CXCL10 may explain the lack of benefit for treatment with oral steroid, albeit at a lower dosage than used in HAM/TSP, in our case. Raised serum β2M and HTLV-1 PVL >1% in blood and higher in CSF, as well as elevated concentrations of neopterin and CXCL10 in CSF support the notion of a putative association between cerebellar ataxia and HTLV-1. Our findings have to be replicated in other ataxia and spastic ataxia cases associated with HTLV-1, in addition more neuropathological studies are warranted in order to characterize the neuroanatomic correlations to motor dysfunction.

**Appendix: Authors**

| Name | Location | Role | Contribution |
| --- | --- | --- | --- |
| Karolina af Edholm, MD | Danderyd’s Hospital, Stockholm | Author | Analysis and interpretation of data, drafting and revising the manuscript |
| Christer Lidman, MD, PhD | Karolinska University Hospital and Karolinska Institutet, Stockholm | Author | Patient care, interpretation of clinical data; revising the manuscript |
| Tobias Granberg, MD, PhD | Karolinska University Hospital and Karolinska Institutet, Stockholm | Author | Interpretation of neuroimaging data; revising the manuscript |
| Graham Taylor, MD, DSc | Section of Virology, Department of Infectious Disease, Imperial College London, United Kingdom | Author | Interpretation of clinical data; selected CSF analyses, drafting and revising the manuscript |
| Martin Paucar, MD, PhD | Karolinska University Hospital and Karolinska Institutet, Stockholm | Author | Study concept and design; patient care, interpretation of clinical data; drafting and revising the manuscript |

**Acknowledgments**

The authors are grateful to the patient for her kind participation for this report, to Dr Carolina Rosadas de Oliveira and Dr Claire Greiller, Imperial College London for performing HTLV related CSF analyses.

**References**

1. Verdonck K, González E, Van Dooren S, et al. Human T-lymphotropic virus 1: recent knowledge about an ancient infection. Lancet Infect Dis 2007; 7(4):266-81.

2. Cooper SA, van der Loeff MS, Taylor GP. The neurology of HTLV-1 infection. Pract Neurol 2009;9(1):16-26.

3. Araujo AQC, Wedemann D. HTLV-1 Associated Neurological Complex. What is Hidden below the Water? AIDS Rev 2019;21(4):211-217.

4. Iwasaki Y. Pathology of chronic myelopathy associated with HTLV-1 infection (HAM/TSP). J Neurol Sci 1990;96(1):103-23.1.

5. Gracia F, Castillo LC, Larreategui M, et al. Relation between human T-lymphotropic virus type I and neurologic diseases in Panama: 1985-1990. J Acquir Immune Defic Syndr Hum Retrovirol 1995;10(2):192-7.

6. Iwanaga K, Mori K. [A case of HTLV-I associated myelopathy with flutter-like oscillation]. [Article in Japanese]. Rinsho Shinkeigaku 1993;33(1):83-5.

7. Kira J, Goto I, Otsuka M, et al. Chronic progressive spinocerebellar syndrome associated with antibodies to human T-lymphotropic virus type I: clinico-virological and magnetic resonance imaging studies. J Neurol Sci 1993; 115(1):111-6.

8. Sato T, Yagishita N, Tamaki K, et al. Proposal of Classification Criteria for HTLV-1-Associated Myelopathy/Tropical Spastic Paraparesis Disease Activity. Front Microbiol. 2018;9:1651.

**Figure 1 Neuroimaging in a HTLV-1 positive patient with ataxia**

Brain magnetic resonance imaging of the reported patient at age 62 years of age showing diffuse subtle atrophy of the cerebellar hemispheres and vermis. Top row: Axial T2-weighted (left and middle) and T2-weighted fluid-attenuated inversion recovery (right). Bottom row: Coronal T2-weighted (left and middle) and sagittal T1-weighted (right) images.
