# Supplementary material for: Expanding the etiologic spectrum of spastic ataxia syndrome: chronic infection with human T lymphotropic virus type 1
Source: J Neurovirol. 2021 Mar 22;27(2):345–7. doi: 10.1007/s13365-020-00932-2 (PMC8192349; doi:10.1007/s13365-020-00932-2)
Supplement: Supplementary file 3 [file 13365_2020_932_MOESM1_ESM.odt]

Cerebellar abnormalities in a patient infected with human T lymphotropic virus type 1 

Additional data

Genetic screening for the most genetic ataxias included in this case tests for spinocerebellar ataxia 1, 2, 3, 6, 7 and dentatorubropallydoluysian atrophy (DRPLA), The autoantibodies analyzed in this case were GAD, onconeuronal, rheumatological and transglutaminase antibodies. Due to increased tremor at rest, a dopamine transporter (DAT) single photon emission computerized tomography (SPECT), was ordered yielding normal results. 

Additional references

e-1 Sato Y, Honda Y, Ohshima Y, Honda E, Oizumi K, Takeuchi N. Acute myelopathy and cerebellar signs associated with uveitis with positive serum and cerebrospinal fluid antibodies to HTLV-I. Kurume Med J. 1994;41(4):193-7.

e-2 Waragai M, Takaya Y, Hayashi (1995). [HTLV-I associated myelopathy (HAM) with cerebellar atrophy presenting a down-beat nystagmus]. [Article in Japanese]. Rinsho Shinkeigaku;35(5):549-52.

e-3 Carod-Artal FJ, del Negro MC, Vargas AP, Rizzo I (1999). [Cerebellar syndrome and peripheral neuropathy as manifestations of infection by HTLV-1 human T-cell lymphotropic virus]. [Article in Spanish]. Rev Neurol;29(10):932-5.

e-4 Fujiki N, Oikawa O, Matsumoto A, Tashiro K (1999). [A case of HTLV-I associated myelopathy presenting with cerebellar signs as initial and principal manifestations]. [Article in Japanese]. Rinsho Shinkeigaku;39(8):852-5.

e-5 Castillo LC, Gracia F, Román GC, Levine P, Reeves WC, Kaplan J (2000). Spinocerebellar syndrome in patients infected with human T-lymphotropic virus types I and II (HTLV-I/HTLV-II): report of 3 cases from Panama. Acta Neurol Scand 101(6):405-12.

e-6 Taki M, Nin F, Hasegawa T, Sakaguchi H, Suzuki T, Hisa Y, Azuma Y, Nakagawa M (2011). A case report of HTLV-I associated myelopathy presenting with cerebellar ataxia and nystagmus. Auris Nasus Larynx;38(3):411-4. 

e-7 Toledo-Cornell C, Santos S, Orge G, Glesby MJ, Carvalho EM (2014). Soluble IL-2 receptor and beta-2 microglobulin as possible serologic markers of neurologic disease in HTLV-1 infection. J Med Virol;86(2):315-21.
